# Supplementary figures and images for: Artificial intelligence algorithms predict the efficacy of analgesic cocktails prescribed after orthopedic surgery
Source: PLoS One. 2023 Feb 2;18(2):e0280995. doi: 10.1371/journal.pone.0280995 (PMC9894442; doi:10.1371/journal.pone.0280995)

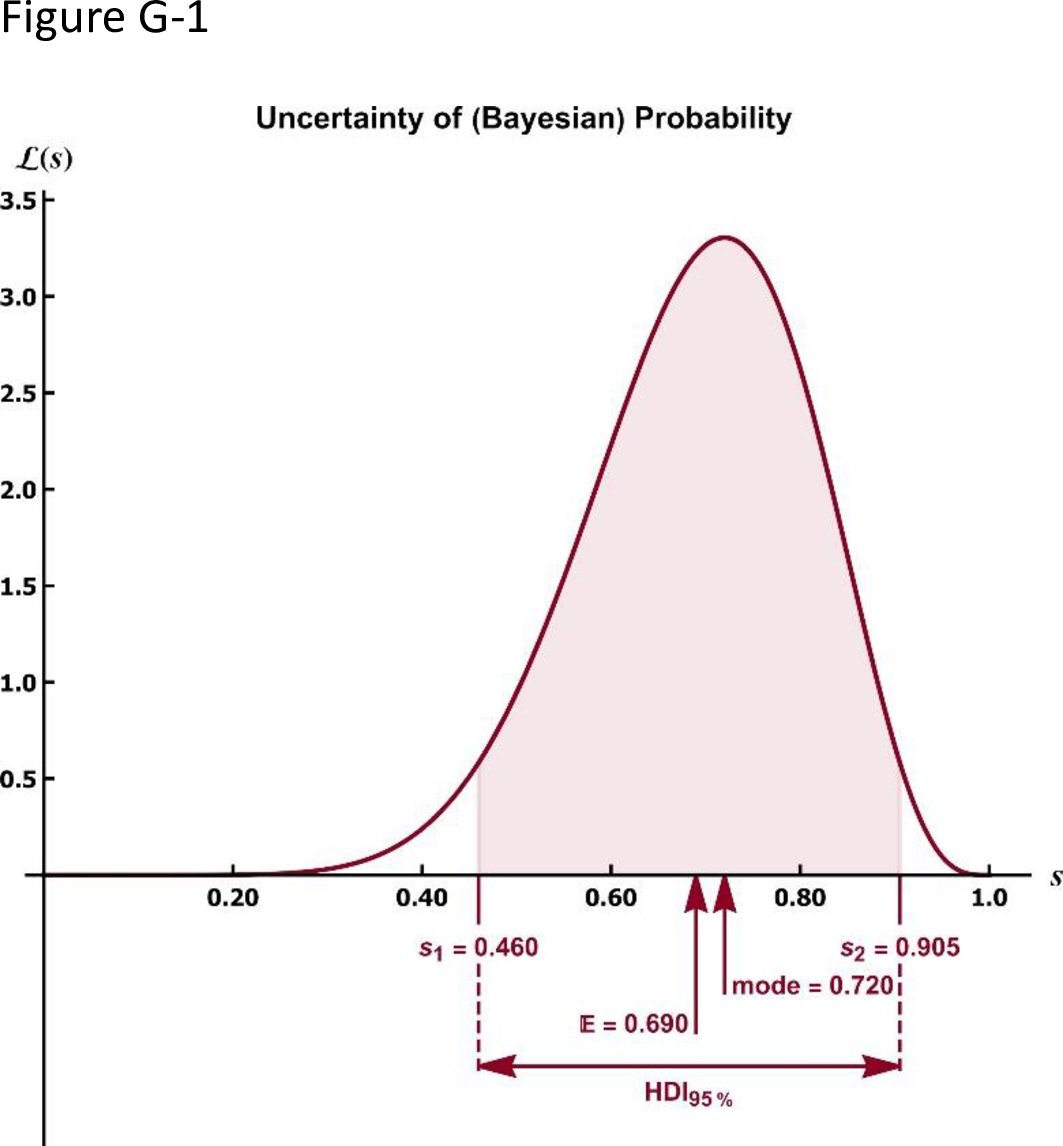

Supplement: S1 Fig — For ease and clarity of description, we show the pdf (the likelihood function Λ(s)) of a Beta distribution, namely that of a Bayesian likelihood. The maximum likelihood is the mode, and the expectation is Ε. The confidence interval HDI95% is shown via a double-ended arrow. The likelihoods at the ends (s1 and s2) of the confidence interval are equal: Λ(s1) = Λ(s2). The shaded area is 95%. (TIF) [file pone.0280995.s001.tif]
