# Supplementary material for: Artificial intelligence algorithms predict the efficacy of analgesic cocktails prescribed after orthopedic surgery
Source: PLoS One. 2023 Feb 2;18(2):e0280995. doi: 10.1371/journal.pone.0280995 (PMC9894442; doi:10.1371/journal.pone.0280995)
Supplement: S1 File — (DOCX) [file pone.0280995.s003.docx]

**GLOSSARY**

**Feature vector extraction**

Consider a patient being administered a cocktail of three analgesics (A_1_, A_2_ and A_3_). If the patient has been administered A_1_ and A_3_ (but not A_2_), how does one encode this for subsequent analysis? For each analgesic, create a 2D vector, with $\left( \begin{matrix} 1 \\ 0 \end{matrix} \right)$ if the analgesic has not been administered, and $\left( \begin{matrix} 0 \\ 1 \end{matrix} \right)$ if the analgesic has been administered. Each of these (three) vectors are pairwise orthogonal (their scalar product is zero) and normalized (their norm is 1) so they are (a) mutually exclusive and (b) introduce no spurious metric into the subsequent analysis. For this patient, there are thus 3 such 2D vectors: $\left( \begin{matrix} 0 \\ 1 \end{matrix} \right)$, $\left( \begin{matrix} 1 \\ 0 \end{matrix} \right)$, and $\left( \begin{matrix} 0 \\ 1 \end{matrix} \right)$, which can be concatenated into $\left( \begin{matrix} \begin{matrix} 0 & 1 \end{matrix} & \begin{matrix} 1 & 0 \end{matrix} & \begin{matrix} 0 & 1 \end{matrix} \end{matrix} \right)^{T}$ . This resulting vector (called a feature vector) is now not necessarily orthogonal to the feature vector of some other patient (who has, say, been administered analgesics A_1_ and A_2_ but not A_3_; the feature vector for the latter patient would be $\left( \begin{matrix} \begin{matrix} 0 & 1 \end{matrix} & \begin{matrix} 0 & 1 \end{matrix} & \begin{matrix} 1 & 0 \end{matrix} \end{matrix} \right)^{T}$ ). In the study we present here, there are 8 different analgesics from which the clinician can choose a cocktail, so the feature vector would be 16-dimensional. Now, consider the pain levels reported by a patient, on an 11-point scale. Because the pain levels are actually categorical variables, the feature vector for a reported pain level would be 11-dimensional with only one nonzero component (the pain level reported). In this study, there are 2 reports of pain levels and therefore two pain feature vectors, each 11-dimensional. Concatenating all the above feature vectors for each patient results in a $2\times8+11+11=38$-dimensional feature vector for each patient. The 750 feature vectors of the 750 patients are *neither* orthogonal *nor* orthonormal and the algorithm methods described below are used to find interdependencies.

Neural networks (NNs)

Neural networks (NNs) are one of the workhorses of AI (Artificial Intelligence). In this glossary, we restrict ourselves to describing how we used NNs for this study. The NN consists of layers; each layer has a sequence of inputs, called nodes (in our case 38 nodes for the first layer) and $n_{2}$ outputs (the number $n$ is the number of nodes in the layer and the index “2” is for the second layer). The outputs of the nodes in first layer are processed from the inputs, but the outputs from one node connected to the nodes of the second layer can vary, depending on the node receiving the processed output of the input layer. The outputs from the second layer are then inputted into a third layer with $n_{3}$ nodes. All processing (‘number crunching’) is the result of mathematical operations. After a certain number of layers, the output is used for further computing, statistical analyses, etc. How many layers to use? How many nodes per layer? These are research questions that are addressed by highly competitive teams (that do not reveal their methods, as they cannot be patented). As is well known, AI using NNs are extremely powerful and used in many lucrative scenarios. The way to adjust the mathematical operations is to train the network. A subset of the data (called the training set) is used to train the outputs until some pre-defined criterion is met (this is called the learning stage). Then the rest of the data (called the test set) is used to evaluate how well the NN has learned. Further training/test cycles are needed (usually far more than 10000) until learning is satisfactory. Because the output criteria are pre-defined, this method of NN learning is called supervised learning.

**Dimension reduction via an autoencoder**

In this study, we cannot use a predefined output — it is unknown. We need unsupervised learning. A modification of the NN architecture consists of many layers, as before, but the output of the last layer is compared with the input of the first layer. If the two agree, based on some rigorously defined statistical criterion, the NN has learned without supervision; this is called unsupervised learning. We use a NN with seven layers, and the innermost layer consists of two nodes. The outputs of this innermost layer are the (two) coordinates of the dimension-reduced feature vector; in other words, this neural network (called an *autoencoder*, because the input of the first layer trains the output of the last layer by checking for agreement) is unsupervised and supplies us with a dimension reduction. The autoencoder therefore learns by detecting interdependencies between the feature vectors of the different patients and the interdependencies of the components of each feature vector without being trained via a pre-specified output. Dimension reduction using autoencoders can and do make unforeseen discoveries.

**Clustering algorithms and DBSCAN**

Clustering is a method of finding (sub)sets (called clusters) in a data set that contain elements that have something in common and are sufficiently different from elements in a different cluster. There exists a large suite of clustering algorithms, because there exists no general algorithm that is suitable for implementation in all applications. We chose DBSCAN (“Density-based spatial clustering of applications with noise”^4^) because it is most suitable for the clustering problem we are confronted with in this study: (a) DBSCAN allows for “background” elements (elements in regions of low density) to be considered noise and does not require these to be assigned to one particular cluster, and (b) it is an unsupervised clustering algorithm — we need/may not specify how many clusters are to be found in the point set. DBSCAN is the most-used clustering algorithm in scientific applications because of these properties. DBSCAN generates clusters by finding points that have many nearby neighbors, based on a user-chosen criterion (usually a distance function) and considers points that are in low-density regions as elements of an outlier cluster. Application of DBSCAN in our study performs exactly as advertised: it finds clusters (which, we discover, are clusters of similar cocktails — i.e. having several analgesics in common) and outputs a “noise cluster” of rarely occurring cocktails that *also* have marginal effectiveness with regards to pain suppression.

**Confidence interval for asymmetric distributions: HDI_95%_**

HDI-95% (oftentimes also labelled HDI_95%_) is “Highest Density Interval at 95% level confidence”. This is — arguably — the most sensible estimator of the uncertainty of a variable, parameter, and/or (Bayesian) probability. (The probability in the Laplace paradigm is a (fixed) number and not a variable; it therefore has no uncertainty.) To be more specific: consider a random variable which is normally distributed. The 95% confidence about the mean (which is, at the same time, the mode) is $\pm1.96s$, where $s$ is the estimated standard deviation, estimated from the data. If a random variable is not normally distributed, then the uncertainty interval cannot be estimated using the above estimator, because, among other matters/issues, the uncertainty is not symmetric about the ML (most likely) estimator (the mode). For non-symmetrically distributed random variables, the mode is much more crucial than the mean, and it cannot be estimated “directly” from the data (i.e., there is no formula for finding it by using the data points). The 1^st^ task is to find the ML distribution of the data; the 2^nd^ task is to find the mode of the ML distribution; the 3^rd^ task is the most challenging: finding an interval about the mode that has, at its two ends, the same likelihood and covers 95% of the *pdf* (the likelihood function) ^17^. S1 Fig shows the relations between the derived properties (mode, expectation, HDI_95%_) for an artificial data set.
