# Supplementary material for: Artificial intelligence algorithms predict the efficacy of analgesic cocktails prescribed after orthopedic surgery
Source: PLoS One. 2023 Feb 2;18(2):e0280995. doi: 10.1371/journal.pone.0280995 (PMC9894442; doi:10.1371/journal.pone.0280995)
Supplement: Attachment — Submitted filename: PONE-D-21-40117R3_response_to_reviewers.pdf [file pone.0280995.s005.pdf]

PONE-D-21-40117R3

Response to the reviewers Salzburg, Nov 21st, 2022

The reviewer either seems to expect a different paper, or has not managed to ascertain what we have discovered and what we communicate. This paper is NOT a paper describing a "how-to" list of procedures. It is a clinical study. A clinical study evaluates (and cautions, if need be) what strategies, procedures, etc. have been undertaken and uses statistical methods to justify the evaluation.

We have written, in our first rebuttal, extensively how AI can evaluate in a novel way. We have, also, listed all kinds of caveats. We do not find it fair that a reviewer who seems to be unwilling to acknowledge (and maybe misunderstands or is not cognizant of) modern methods that improve the evaluation methodology of clinical studies. We have clearly written about many studies not taking the "curse of dimensionality" into account. We have performed dimensionality reduction for this very reason. This reviewer seems to want additions of more and more variables, thus increasing the "curse of dimensionality" rather than acknowledging our success in overcoming this "curse". We find, and present in the paper, that dimension reduction delivers a remarkable insight of the effectiveness of some cocktails.

Surprisingly, we have introduced many further variables, but not the ones clinicians are used to introducing. We have introduced many, many combinations of pain killers (we call them cocktails). As we pointed out, not only in the manuscript, but in our first rebuttal, that we are evaluating pain relief, and how one cocktail is overwhelmingly successful at it.

We repeatedly ask the reviewer to acknowledge that we are evaluating the effectiveness of pain cocktails, and performing dimension reduction on these many combinations. To repeat: we are unsure whether this reviewer really appreciates our achievement of dimension reduction. And it is somewhat derogatory, to belittle our success by describing pain levels (which are categorical variables, not numbers) as "just numbers".

We are surprised that Reviewer #1 does not appreciate that this is an evaluative clinical trial.

We would also like to be frank with you, the editor. Reviewer #1 is not familiar with modern statistical methods, and thus seems to fall victim to the paradigm of "what to prescribe as pain medication for the following set of surgeries". This doesn't work, as modern methods used for controlling for the "curse of dimensionality" have repeatedly shown.

We submitted this manuscript to PlosONE because we were under the impression that this journal is interested in (a) progress, (b) modern methods of evaluation applied to clinical study methodology, and (c) discovery of statistical effects that a conventional study plan cannot find.

Ad (c): We claim that a conventional study plan would not discover what we present in this manuscript. There are far too many surgery parameters and far too many pain medication cocktails. Not only would such a setup require several thousand patients, but even then the “curse of dimensionality” (to belabor the point again) would not give a reliable signal. Many papers published along traditional lines “cut corners” in their analyses, especially when they use statistically discredited methods, such as factor analyses or SEM (because these methods rely on mapping the pain levels into computable integers).
