# Supplementary material for: Artificial intelligence algorithms predict the efficacy of analgesic cocktails prescribed after orthopedic surgery
Source: PLoS One. 2023 Feb 2;18(2):e0280995. doi: 10.1371/journal.pone.0280995 (PMC9894442; doi:10.1371/journal.pone.0280995)
Supplement: Attachment — Submitted filename: PONE_answers_to_the_reviewers.docx [file pone.0280995.s004.docx]

Rebuttal Letter

Specific comments:

1. line 21 consider "potential" instead of "claimed" - efficacious synergy has been established.

Thank you, this has been changed in the manuscript.

2. line 46 - be specific about certain aspects of orthopaedic surgery - particularly the interest in "outpatient" total joints in the US, extremity trauma, and shoulder surgery (e.g. rotator cuff repair) are the most painful orthopedic surgeries. This is where pain control is most needed.

In our revision we have specified the most relevant procedures for adequate pain control.

3. line 50 - This sentence requires a little more explanation. What specific efficacy are you speaking about?

We thank the reviewer for this comment since the complexity of pharmacological interactions of different drugs represents the major aspect of our work. In the respective paragraph we have inserted some examples of pharmacological properties of the investigated pain medications. This is thought to serve as an explanation for the readers

4. It would be useful to in 1 or 2 sentences explain why conventional logistic regression cannot answer this question. Those unfamiliarwith ML will not understand this.

Conventional logistic regression occurs in two types: binomial and multinomial. In both, the explanatory variable must be a continuous random variable. In the case of binomial logistic regression, there is no problem with the predicted/explained variable; in the multinomial case, the statistics of the explained random variable are not statistically ‘clean’. The difficulties in the multinomial regression are usually glossed over, with much hand-waving. If logistic regression is to be used, and the dependencies are highly nonlinear, then the regression requires many nonlinear terms, such as products; the ‘curse of dimensionality’ kicks in. There exists, in the case of multinomial regression no criterion for limiting the terms in the explanatory variables. True, one could use Akaike’s Information Criterion, but this criterion is derived for the Laplace limit of infinite sample size. Even if one were to apply Takeuchi’s correction, then one would not know which of the nonlinear terms one would have to ‘sacrifice’.

In the analyses we present in this manuscript, we avoid all these problems. All random variables are categorical, so the usual regression doesn’t apply. Secondly, we cannot (because of the nature of the problem, not because of our inability) distinguish between predictor and predicted variables. Unsupervised neural networks ‘naturally’ overcome (not: eliminate) the curse of dimensionality. In addition, the way we have set up the analysis, we need worry about random variables being categorical, and all nonlinearities are taken care of (by the cutoff criterion at each of the edges in the neural network).

5. Line 59 – the first part of this sentence is confusing. Specifically the part “to learning algorithms labelled AI (artificial intelligence); the latter”. You could get rid of that and the sentence is less confusing to read for the novice. ML really come sin 3 types – supervised, unsupervised, and reinforced learning. IN geneerallines 59-67 don’t add much to this introduction. When you talk about unsupervised neural networksit isn’t clear how the “outputmimics the input”. For example supervised ML is more task driven problems, unsupervised is more data driven type problems. Can you give an example of how the output mimics the input.

We are grateful to the reviewer for these comments. We do want to point out that, in this manuscript, are dealing with AI, which is not ML (machine learning), although there are overlaps. We leave out the ML aspects in our methodology and restrict our description to unsupervised NN (in essence: dimension reducers). We have included both a graph showing how the terminology of autoencoders relates to the one-hot encoded input feature vectors and the dimension-reduced coder outputs.

We have modified this part of the text.

6. line 68 - "We elaborate:" isn't common phrasing.

Has been deleted

7. lines 70-73 - A figure or two would make this much easier to understand. If the goal of the paper is to help the clinician understand and appreciate (and believe) what ML can do for clinical medicine they will not understand this or likely take the time to try and understand it.

We have added a figure and a lengthy figure caption.

8. line 87 what is the "crossed" anterior ligament. Please, provide a table with the exact number of patients getting specific procedures. Because not all of these surgeries are equally as painful and pain cocktails used in spine surgery will inherently differ from those used in TJA. So this is potentially confounding.

We apologize for the confusion. Although our manuscript has undergone language editing this has been overseen. A table will be provided as part of the manuscript. We want to point out that in our investigation we did not use absolute pain values but the difference between maximum and minimum values which fairly represents pain reduction.

9. was this data collected retrospectively or prospectively or a retrospective analysis of a prospective database?

The study is a retrospective analysis of prospective collected data. A remark within the text has been made.

10. Any study of pain medicine and the efficacy should include any preoperative narcotic or controlled substance use as this can have major effects on postoperative pain medicine utilization. Was there any attempt to quantify or control this?

Indeed, we have made a chart review and only found 4 patients with a chronic use of opioids.

11. Line 102 its about 50/50 general to regional anesthesia. Its important to note if the is neuroaxial (spinal/epidural), or peripheral nerve blocks like femoral nerve or saphenous nerve or iPACK for TKA. This is critical information along with the drugs that were used in the blocks. They all have different onset and duration times which will affect the utilization and efficacy of the scheduled pain meds and will differ between surgeries, surgeons, and anesthesiologists.

We agree with the reviewer’s point of view. The authors are fully aware of the different analgesic properties of the used methods. Nonetheless we only investigated the pain-reducing capacities of analgesic cocktails.

12. line 105 - please clarify points "a" what is "maximum flexing" - this sounds painful.

Thank you for the remark. The text is misleading at this point. We have completed the text with the term “pain” in order to eliminate this misunderstanding

13. Were NRS only collected until 2pm on POD 1?

Yes.

14. Table 1 – what is HDI 95%.. Why is there so much descriptive detail about the patients’ ages. Age is but just one variable in an orthopedic study.

(a) HDI-95% (oftentimes also labelled HDI_95%_) is “Highest Density Interval at 95% level confidence”. This is — arguably — the most sensible estimator of the uncertainty of a variable, parameter, and/or (Bayesian) probability. (The probability in the Laplace paradigm is a (fixed) number and not a variable; it therefore has no uncertainty.) To be more specific: consider a random variable which is normally distributed. The 95% confidence about the mean (which is, at the same time, the mode) is $\pm1.96s$, where $s$ is the estimated standard deviation, estimated from the data. If a random variable is not normally distributed, then the uncertainty interval cannot be estimated using the above estimator, because, among other matters/issues, the uncertainty is not symmetric about the ML (most likely) estimator (the mode). For non-symmetrically distributed random variables, the mode is much more crucial than the mean, and it cannot be estimated “directly” from the data (i.e., there is no formula for finding it by using the data points). The 1^st^ task is to find the ML distribution of the data; the 2^nd^ task is to find the mode of the ML distribution; the 3^rd^ task is the most challenging: finding an interval about the mode that has, at its two ends, the same likelihood and covers 95% of the *pdf* (the likelihood function).
(b) Below, we supply a graphic for this. We are unsure whether we can include a graphic in the manuscript. Use of HDI-95% is standard in Bayesian statistics; we list a textbook that can be referenced (J.K. Kruschke *Doing Bayesian Data Analysis. A Tutorial with R, JAGS, and Stan.* Academic Press, London, UK, 2015).

We need to test whether age is a confounding variable. We have done so. Use of KDE and HDI_95%_ provides strong evidence that age is not a confounding variable. We have pointed this finding out in the manuscript.

15. Line 195 - this just drops off, its an incomplete sentence.

Thank you for pointing this error out to us. We have deleted this incomplete sentence.

16. line 235 -. Generally summarize what you found in 1 paragraph, 2nd paragraph how is it the same with what is in the literature, 3rd paragraph how is it different than what is in the literature, 4th - what other interesting variables that you found that have never been looked at before, 5th limitations, 6th conclusion.

We have done so. Thank you for your recommendation.

17. Please, clarify the purpose and the implications of the autoencoder part of the analysis. Does the resultant two-dimensional representation of the input data contain any generalized knowledge or does it just directly encode the input?

An autoencoder just directly (re)encodes the input. This may seem to imply that no generalized knowledge becomes available — not true. By multiplying the output of one layer with matrices to generate the input of a further layer, and multiplying these with some threshold function (usually a sigmoid), it produces a dimension reduction in a most notable way: it finds interrelationships between the components of the input vectors. By including a threshold function, such as a sigmoid, nonlinearity is included. In contrast to factor analysis, say, the interrelations are not necessarily linear combinations of the inputs.

Any autoencoder is an unsupervised neural network. It does not generate an anticipated output, and therefore its reliability cannot be tested against some expected output. Please observe that this derivation of relationships is different from the use of supervised neural networks that supply a probability of an output classification. The (unnerving — for humans) feature of an autoencoder is its ability to detect interrelations between the input components, but these are only revealed as clusters in the (encoded) dimension-reduced feature vectors. Another way of phrasing this state of affairs: if the autoencoder succeeds in dimension-reduction, then the outputs within clusters have interrelationships within their input components that are different from the interrelationships for other input vectors that are outputted to different clusters in the dimension-reduced output space.

The encoding accuracy, e.g. the measured by the variance explained, is also not reported.

We argue that your description of how neural networks function/operate infers a different approach, one that is grounded on metric variables. Artificial neural networks that are autoencoders seek to decrease the entropy, usually by minimizing the square of the difference between the sum of the squares of the input vectors and the sum of the squares of the output vectors. (These sums of squares should be equal, because the output is to mimic the input.) In particular, for the analysis we present in this manuscript, there is no variance to be explained, because the inputs are categorical/ordinal variables.

It is therefore not unlikely that the clusters encode the most abundant combinations of analgesics in cocktails whereas the X and Y coordinates in the clusters encode the pre-and post-analgesia pain levels, which may be correlated.

As noted above, there are no correlations. Please allow us to point out that it is common knowledge that high correlations do not infer causality, but also that correlations of zero (0) do not infer independence. It seems to us that discussing correlations is one from a different paradigm.

This can be tested via encoding the analgesics cocktail components without corresponding pain levels and seeing whether the five clusters are formed again.

As mentioned, because the autoencoder seeks (via nonlinear interrelationships) structure in the output, driving the autoencoder with the analgesics alone (i.e. without pain levels) and then analgesic cocktail components with the pain levels are not comparable dimension-reduction encodings. “Doing” the encoding separately finds other interrelationships.

Third, there seem to be no conclusions derived from the obtained clusters. What would belonging to a certain cluster mean except for a high overlap in the list of the cocktail's components?

In a certain, overly rigorous interpretation of our presented results (which we welcome and are grateful for, by the way), the reviewer’s criticism is well placed. Neural networks (autoencoder or otherwise) do not infer more than the existence of clusters (or not: for some data sets, clusters cannot be found; then the input data has not structure). Indeed, the clusters we found “discover” a high overlap. We cannot infer any (analgesic) mechanism that generates the overlap. Please allow us the following (granted, simplistic) summary: these statistical methods apply artificial intelligence. That said, we (humans) cannot claim to understand the output. We can only analyze the output.

One way to use this embedding is to predict the pain reduction efficacy and significance for the cocktails not involved in the study via training another network on the two-dimensional embedding of the inputs.

True, we could use the detected embedding to predict pain reduction efficacy by training a further, supervised neural network. We have not done so for two reasons: (a) we would need a vastly larger data set (it is already difficult enough to find the clusters with the 750 data points we have, because there are so many cocktails), and (b) a discussion of introducing the testing with a further, supervised neural network would have made the manuscript even lengthier and would let it drift away from the analgesic aspect into a manuscript about how to best implement the “Titanic challenge”.

Finally, no argument is presented as to why autoencoder was used instead of simpler yet more interpretable alternatives such as CMDS, MDS, or Isomap.

MDS (multi-dimensional scaling) is a dimension reduction algorithm which analyzes the dissimilarity matrix. Because we have feature vectors derived from ordinal/categorical variables, we cannot define dissimilarity. If one were to construct a dissimilarity matrix so as to use MDS, we would have to map the categorical variables into cardinal numbers — which is a fallacious procedure (Blalock, 1960).

We are unaware of the algorithms CMDS. Our reference list contains a text Sun, Ji, Ye *Multi-Label Dimensionality Reduction*, CRC Press, 2014) which covers a very large number of dimension reduction algorithms, but we were unable to find this acronym.

The algorithm Isomap is a Python version of dimension reduction using an autoencoder. In fact, the webpage <https://benalexkeen.com/isomap-for-dimensionality-reduction-in-python/> describes the dimension reduction of the Swiss roll in exactly the same way as does the webpage <https://www.wolfram.com/language/11/neural-networks/learn-a-parameterization-of-a-manifold.html> for MATHEMATICA from Wolfram Language, the software package which we use. If Isomap from Python is considered simple and interpretable, then our result obtained with the (programmable) autoencoders from Wolfram Technologies must be considered equally interpretable. One further comment, please: the example presented in Ben Keen’s webpage (URL above), is exactly a dimension reduction application which the statistician in the author list has used in a publication for human faces expressing fear and apprehension. The reason we chose MATHEMATICA is: proprietary packages have better (in the sense of being more time and memory-demands efficient) than publicly available ones.

We are hesitant to make extensive comments about dimension reduction and specify why we did not consider or implement the 20+ different dimension reduction algorithms. We argue that inclusion of such a description would distract from our using the currently most powerful algorithms for this type of medical data.

Minor comments:
Please change "ML statistics" to "statistics", and "AI" to "ML".

We have reviewed the manuscript. Unfortunately, ML in the world of statistics also means “maximum likelihood”. In all the sentences in which we use “ML”, we mean maximum likelihood. We are aware that “ML” can also mean “machine learning”. Because machine learning is an overarching terminology (which usually implies those aspects of machine learning that are not based on neural networks — supervised or unsupervised), we avoided the use of ML in the “machine learning” sense in the manuscript. For the presentation in the manuscript, especially those sections that use Bayesian statistics, maximum likelihood is essential, so we would cause confusion if we left out “ML”. On the other hand, we use the “AI” (artificial intelligence) aspect of machine learning, and no other. For this reason, we have not used “ML” in the machine learning sense anywhere in the manuscript.

Explain the choice of parameters for DBSCAN. For example, it seems reasonable to split cluster #2 into two clusters and to merge clusters #5 and #6 into one cluster.

We do not (and cannot) choose parameters for DBSCAN. DBSCAN is a “a density based non-parametric algorithm” (quote from WIKIPEDIA). None of the implementations of this clustering algorithm that we are aware of allows the introduction of, and the choice of, parameters.

Your observation about how the clustering output of DBSCAN is seemingly unnerving is a perception we share with you, the reviewer. That is an outcome of how DBSCAN works. We cannot override its output, unless we discard the algorithm. The statistician in the author list has encountered numerous such situations in his daily work.

Captions for Fig.1 and Fig. 2 appear swapped.

Thank you for pointing out this error.

Contrary to the claim in the paper, cocktail choices are not a random (as in: i.i.d.) variable.

Indeed, cocktail choices are random variables. They are not continuous random variables, and certainly not iid random variables (as in ordinary LSq regression, for example).

Please explain why the abundance of common ingredients in the same clusters deems surprising.

Any result that cannot be anticipated and revealed as a result of an unsupervised NN is surprising. The NN is unsupervised. If it outputs an anticipated result, then it is surprising (for us) that it did so; if it outputted a result that we did not anticipate, then it is surprising.
